# Suicidal expressions among young people in Nicaragua and Cambodia: a cross-cultural study

**DOI:** 10.1186/1471-244X-12-28

**Published:** 2012-03-31

**Authors:** Claudia Obando Medina, Bhoomikumar Jegannathan, Kjerstin Dahlblom, Gunnar Kullgren

**Affiliations:** 1 Centre for Demography and Health Research, Nicaraguan National Autonomous University, León, Nicaragua; 2 Center for Child and Adolescent Mental Health (CCAMH), Takhmau, Cambodia; 3 Department of Public Health and Clinical Medicine, Division of Epidemiology and Global Health, Umeå University, SE-901 85 Umeå, Sweden; 4 Department of Clinical Sciences, Division of Psychiatry, Umeå University, SE-901 87 Umeå, Sweden

**Keywords:** Suicidal expressions, Adolescents, Young People Cross-cultural comparison, Nicaragua and Cambodia

## Abstract

**Background:**

Whereas prevalence of suicidal expressions among young people is fairly similar in different countries, less is known about associated risk factors. This study compares young people in Nicaragua and Cambodia to examine if the pattern of association between mental health problems and suicidal expressions differs.

**Methods:**

368 and 316 secondary school students, from each country respectively, participated. Self-reported suicidal expressions, exposure to suicidal behavior in significant others and mental health problems among the students were measured using Attitude Towards Suicide (ATTS) and the Youth Self-Report (YSR) questionnaires.

**Results:**

Prevalence of serious suicidal expressions (plans and attempts) during recent year, did not differ between countries. Cambodian young people scored significantly higher on all eight YSR-syndromes, except for withdrawn/depressed. In Nicaragua, all YSR-syndromes were significantly associated with serious suicidal expressions in both genders compared to Cambodia where only one syndrome showed an association in each gender; Withdrawn/depressed among girls and Somatic complaints among boys. Associations between being exposed to suicide among significant others and serious suicidal expressions also differed between Cambodia and Nicaragua.

**Conclusions:**

While the magnitude of serious suicidal expressions is similar between these structurally similar but culturally different countries, determinants behave differently. Qualitative studies are warranted to further explore cultural specific determinants for suicidal expressions among young people.

## Background

Understanding suicide attempts and other related suicidal expressions is essential to improve mental health and to prevent suicide among young people. A review of population based studies estimated that a mean proportion of 9.7% of adolescents report a life-time suicide attempt [1]. Even though not all attempts are motivated by a wish to die the attempt still represents the strongest single risk factor for a suicide and by its own is a strong indicator of severe mental distress [2,3]

Background and meaning of suicidal expressions differ between cultures and cross-cultural studies leads to a deeper understanding of suicidal expressions among young people [3]. For example, socio-economic factors or mental health problems influence suicidal behavior differently in different countries [4,5] and religion might be a protective factor for suicidal expressions in some countries and in others not [6].

Furthermore, the gender differences in suicidal expressions must be taken into consideration in cross-cultural studies. For completed suicides, there is a male dominance in most countries with exception in some Asian countries [7,8] but for suicide attempts and other suicidal expressions the gender pattern is different and more complex. In hospital-based samples, girls predominate whereas community based samples show few gender differences. For example, a Nicaraguan hospital based survey showed that suicide attempts were more than three times as common among girls as compared to boys [9], whereas a community-based study in the same setting showed no significant gender differences in self-reported suicide attempts [10]. Gendered cultural beliefs influencing help seeking behavior might be one explanation, similar to what has been reported for young American Indians [11].

Most cross-cultural studies have been from middle or high-income countries [4,12] and to our knowledge there is no study that compares suicidal expressions among young people in relation to established risk factors such as mental health problems and exposure to suicide between low-income countries from different continents.

This study compares Nicaragua and Cambodia, two countries that have been through civil war, extreme social and political turmoil and share common structural conditions and widespread extreme poverty. In Nicaragua, suicide rates among young people are the highest among all Latin and Central American countries [13]. There are no reliable figures on suicide rates in Cambodia. Despite these structural similarities, there are also cultural differences. By comparing these two countries, we hope to throw new light on suicidal expressions and their determinants among young people.

## Method

### Definitions

The nomenclature in the field of suicidology "perennially dissatisfies researchers and clinicians", as De Leo and co-authors put it in their overview article [14]. In the present study, we use a term that most researchers seem to be satisfied with--suicidal expressions which cover a spectrum of suicide related phenomena except completed suicide. It includes life weariness, death thoughts, death wishes, suicide ideation, suicide plans and suicide attempts.

### The settings

Nicaragua and Cambodia share similar demographic characteristics and both are ranked as "Medium human development" countries in the Human Development Index 2010 (117 and 126 rank respectively). Though Nicaragua is located in Central America and Cambodia in Southeast Asia, both are located in the tropical zone between 10 and 15 degrees north of the equator.

Poverty is a major concern as 16% of the population in Nicaragua and 37% in Cambodia live under absolute poverty (< 1 USD per day) [15,16]. Cambodia is less urbanized as 80% of the population live in rural areas when compared to 41% in Nicaragua.

In both countries the population is young. In Nicaragua 35% (median age 22) of the population of 5.7 million and 33% of 14.8 million in Cambodia are below 15 years (median age 22) [17,18].

Adult literacy rates are estimated to be 78% in both countries, and mean years of schooling are around 6 years [19]. In Nicaragua, the level of education remains the lowest in Central America and 72% of the populations do not complete secondary education. Eighty percent of students attend public schools. Drop-out rates are high, and it takes an average of 10 years to complete 6 years of primary school [20].

In Cambodia, the formal educational structure consists of six years of primary school, three years of secondary school, and three years of higher secondary school. For every one thousand students who enroll in primary school, only 27 graduate from upper secondary school [21].

Young people in Nicaragua and Cambodia face similar health challenges, such as sexual abuse, drug abuse and reproductive health problems--all factors correlating with suicidal behavior [22].

Both Cambodia and Nicaragua suffered violence and social turmoil but of different nature. In Cambodia, the trauma of the 'Pol Pot era' has left scars in the parents of today's young people. Nicaragua has likewise had a history of political instability and civil war but without the extreme collective trauma that characterizes the Cambodian situation [23].

In both countries, family relationships are highly valued and include relatives beyond the nuclear family unit even though family structure in Nicaragua is more dismantled, much due to migration [24].

The most obvious difference is religion. In Nicaragua, Catholicism strongly dominates whereas Buddhism is the predominant religion in Cambodia

### Instruments

The **Attitudes towards Suicide (ATTS) **is a self-report questionnaire that covers three areas; (1) Exposure to suicide from significant others such as parents, siblings, friends etc., (2) Attitudes towards suicide and (3) own suicidal expressions. In the present study we used sections 1 and 3. The psychometric properties of the instrument have been reported in previous studies [25-27]. The semi-structured questionnaire was translated to Spanish and Khmer for use in Nicaragua and Cambodia, respectively. The translated version of ATTS was discussed among professionals and field-tested, and had been used in both the countries, previously [10,28,29].

In this study 'Exposure to suicide from significant others' was defined as reported attempted or completed suicide among parents, siblings, partners or friends. Regarding own suicidal expressions, 'Serious suicidal expression' was defined as either plans or attempts during recent year.

The **Youth Self-Report (YSR) **developed by Achenbach and colleagues is a self-report questionnaire for 11-18 year olds that evaluates competencies and behavioral problems. In our study we explored the behavioral part, consisting of 112 items with statements of behaviors or symptoms, including 16 items indicating social desirability. The participants responded as following: 0 = not true; 1 = somewhat or sometimes true; and 2 = very true or often true. The items are combined into eight syndromes: withdrawn/depressed, somatic complaints, anxious/depressed (together constituting the internalizing syndrome), rule breaking behavior and aggressive behavior (together constituting the externalizing syndrome), social problems, thought problems and attention problems [25]. The total problems score is the sum of all the responses comprising of the different syndromes in YSR, whose reliability and validity has been established across diverse cultural settings [10,12,30].

### Study design and sample

We conducted a cross-sectional study among students aged 15-18 years in public schools in both countries during the year 2008-2009.

#### Nicaragua

This study was conducted in the nine public urban secondary schools in Leon municipality where 3162 pupils were enrolled in school during the study period. We used a random stratified sampling technique proportional to the size of each school to select the informants. For the final analyses 188 boys and 180 girls were included.

#### Cambodia

A sample of all the students of randomly selected classes, from level 10 and 11 in the two higher secondary schools took part in the study. The sample size, depending on the size of the class, was arrived at based on the prevalence figures from previous studies in Nicaragua [10]. The two higher secondary schools are located in Takhmau, Kandal province, a semi-urban area, 8 km south-east of Phnom Penh, the capital city of Cambodia. For the final analyses 151 boys and 165 girls were included.

### Ethical considerations

The study was approved by the ethical committee of the UNAN León University, Nicaragua and Umeå University (07-046 M), Sweden. In Cambodia the study was approved by the school-directors and the parents were informed about the study through the parent-teacher association. In both settings, we visited each school and explained the aims of the study to the students as well as to the principals, teachers and the representatives of the educational system. The participation was entirely voluntary and questionnaires were returned anonymously. The researchers informed the participants about the availability of free-counseling services.

### Internal consistency and social desirability

The internal consistency was acceptable for all YSR syndromes in both countries except for attention problems among the girls in Cambodia. For internalizing and externalizing syndromes the reliability was good (Table 1). Very similar reliability coefficients have been reported from, for example, a Swiss study [31].

**Table 1:** Internal consistencies (Cronbach's Alfa) for YSR syndrome scales

	Nicaragua		Cambodia	
	**Boys**	**Girls**	**Boys**	**Girls**

Anxious/depressed	0.68	0.76	0.69	0.72

Withdrawn/depressed	0.59	0.67	0.60	0.62

Somatic complaints	0.72	0.66	0.66	0.67

Social problems	0.66	0.70	0.67	0.54

Thought problems	0.66	0.61	0.66	0.67

Attention problems	0.68	0.70	0.30	0.56

Rule breaking behavior	0.72	0.68	0.60	0.57

Aggressive behavior	0.78	0.76	0.73	0.74

Internalizing symptoms	0.84	0.85	0.82	0.84

Externalizing symptoms	0.85	0.83	0.79	0.78

The social desirability scale contains items like: "I am quite honest", "I like to help others when they need" and "I try to be fair towards others". There was no significant difference regarding social desirability score between Cambodian and Nicaraguan youth.

### Analysis

The missing data on single YSR-items was less than 10% in both countries and the missing values were imputed by the median. Chi -square was used to compare prevalence by country and gender and t- tests were used to compare mean YSR values by country and gender. Uni- and multivariate analyses were used to analyze the association between serious suicidal expressions vs. YSR-syndromes and exposure to suicide. Since validated cut-off points for "casesness" among YSR-syndromes do not exist for Nicaragua or Cambodia, the YSR-syndromes were entered as continuous variables in the logistic regression analyses, i.e. ORs were calculated based on one point increment on YSR-syndrome scores.

## Results

The total sample size was 684 students, 54% from Nicaragua and 46% from Cambodia. 50.4% of the informants were girls and there were no significant differences in gender distribution between countries.

### Prevalence of suicidal expression by gender and country

In both genders, death thoughts, death wishes and suicide ideation and suicide attempts were more frequently reported by young people in Nicaragua than in Cambodia. However, for serious suicidal expressions (plans and/or attempts) there were no significant differences within either gender Table 2.

**Table 2:** Prevalence of confirmatory responses to questions on suicidal expressions during recent year--stratified for gender and country

Suicidal expressions	Boys			Girls		
	
	Nicaragua n = 188 %	Cambodia n = 151 %	p	Nicaragua n = 180 %	Cambodia n = 165 %	p
Life weariness	34.0	26.5	n.s.	34.4	24.8	n.s.

Death thought	36.2	25.8	.042	43.3	26.1	.001

Death wishes	25.0	14.6	.018	33.9	17.6	.001

Suicide ideation	17.6	8.6	.017	27.8	13.3	.001

Plans/attempt	10.1	16.6	n.s.	12.8	12.7	n.s.

Any expression	57.4	54.3	n.s.	63.3	49.1	.008

### Mental health syndromes (YSR) by gender and country

As shown in Table 3, Cambodian adolescents scored significantly higher on almost all YSR-syndrome scales with exception of rule-breaking behavior in both genders and withdrawn depressed and externalizing syndrome among boys. Despite significant country differences, there were few gender differences within each country.

**Table 3:** Mean scores, standard deviation (SD) for YSR-syndromes as related to gender and country

YSR syndrome	Mean (SD)		P	Mean (SD)		P
	**Boys**			**Girls**		

	**Nicaragua** **n = 188**	**Cambodia** **n = 151**		**Nicaragua** **n = 180**	**Cambodia** **n = 165**	

Anxious depressed	**4.3 (2.6)**	**8.2(3.5)**	**0.00**	**5.2(2.9)**	**9.7(4.0)**	**0.00**

Withdrawn depressed	3.9 (1.9)	4.3(2.4)	n.s.	**4.1(2.1)**	**4.9(2.5)**	**0.00**

Somatic Complaints	**3.1(2.3)**	**6.3(3.2)**	**0.00**	**4.0(2.2)**	**6.9(3.2)**	**0.00**

Social Problems	**4.5(2.4)**	**6.4(3.2)**	**0.00**	**4.4(2.5)**	**7.1(2.8)**	**0.00**

Thought Problem	**2.8(2.1)**	**5.3(3.2)**	**0.00**	**2.5(1.9)**	**4.9(3.2)**	**0.00**

Attention Problems	**4.5(2.3)**	**7.6(3.0)**	**0.00**	**4.2(2.3)**	**8.1(2.6)**	**0.00**

Rule breaking Behavior	4.8(2.8)	4.3(2.8)	n.s.	3.1(2.3)	3.1(2.2)	n.s.

Aggressive Behavior	**6.8(3.5)**	**8.4(3.8)**	**0.00**	**6.4(3.2)**	**8.8(4.1)**	**0.00**

Internalizing Syndrome	**11.6(5.8)**	**18.7(7.2)**	**0.00**	**13.6(6.1)**	**21.3(8.2)**	**0.00**

Externalizing Syndrome	11.8 (5.8)	12.7(5.8)	n.s.	**9.5(5.0)**	**11.9(5.3)**	**0.00**

Total problem score	**38.3 (16.5)**	**47.5 (14.1)**	**0.00**	**37.4 (17.2)**	**49.2 (14.7)**	**0.00**

### Exposure to suicide from significant others by gender and country

Total prevalence of exposure to suicide by significant others (parents, siblings, partners or friends) differed between countries. 16.8% in Cambodia and 26.4% in Nicaragua (*X*^2 = ^= 9.126; p = 0.00). Among girls, there were no significant difference in terms of reported exposure between countries, but Nicaraguan boys had higher exposure to suicide by significant others (25.5%) than Cambodian boys (12.6%) (*X*^2 ^= 8.855;p = 0.00)

### Association between YSR-syndromes and exposure to suicide

Table 4 shows univariate analyses within each gender divided for country on YSR-syndromes (entered as continuous variables in the logistic regression model) and exposure in relation to serious suicidal expressions. Among girls in Nicaragua all factors showed a significant association with serious suicidal expressions whereas in Cambodian girls only withdrawn/depressed, internalizing and exposure were associated. This cross-national difference was even more pronounced among boys where all factors were significantly associated with serious suicidal expressions among Nicaraguan boys but only one factor, somatic complaints, showed association among Cambodian boys.

**Table 4:** Crude ORs for YSR-syndromes and exposure to suicide among significant others as related to serious suicidal expressions

	Girls				Boys			
	**Nicaragua**		**Cambodia**		**Nicaragua**		**Cambodia**	

	**OR**	**p**	**OR**	**p**	**OR**	**P**	**OR**	**p**

Anxious/depressed	1.506	.000	1.086	n.s.	1.556	.000	1.083	n.s.

Withdrawn/depressed	1.275	.032	1.328	.002	1.650	.000	1.164	n.s.

Somatic complaints	1.545	.000	1.143	n.s.	1.248	.028	1.189	.016

Social problems	1.363	.001	1.053	n.s.	1.478	.001	1.061	n.s.

Internalizing	1.236	.000	1.064	.023	1.205	.000	1.054	n.s.

Externalizing	1.181	.021	1.044	n.s.	1.156	.001	1.017	n.s.

Total YSR problems	1.066	.000	1.032	n.s.	1.068	.000	1.019	n.s.

Exposure to suicide	2.871	.021	2.793	.040	6.333	.000	2.000	n.s.

(YSR-syndromes entered as continuous variables in the logistic regression analyses)

More from a gender perspective, the pattern of association between factors and serious suicidal expressions was similar between boys and girls in Nicaragua where all factors showed a significant association with serious suicidal expressions. In Cambodia, three of the factors were significantly associated for girls, whereas boys only showed a significant association between somatic complaints and serious suicidal expression.

In multivariate analyses, performed separately for each gender and country, where all factors were entered in a logistic regression model, no significant association remained among Cambodian boys whereas anxious/depressed (OR = 1.108; p = 0.00) and exposure (OR = 4.249; p = 0.01) remained significant among Nicaraguan boys. Among Cambodian girls only withdrawn/depressed (OR = 1.426;p = 0.00) remained and among Nicaraguan girls anxious/depressed (OR = 1.149;p = 0.00) and somatic complaints (OR = 1.390;p = 0.01) remained significantly associated.

## Discussion

The overall aim of the present paper was to examine suicidal expressions and some possible determinants in two very poor post-conflict societies from two different continents to identify differences that might reflect cultural differences. Three findings are of particular interest.

Firstly, this study shows that there is no significant difference in prevalence of serious suicidal expressions in Cambodia as compared to Nicaragua, even though milder expressions differ somewhat. Overall in both countries, prevalence of various suicidal expressions is in the lower range as compared to studies from middle or high income countries [4,32].

Secondly, this study shows that Cambodian youth, both girls and boys, score considerably higher on almost all YSR-syndromes than Nicaraguan youth, for many syndromes with twice as high scores. Several previous studies have reported cross-cultural differences on YSR-scores but mostly to a lesser extent. For example, in a study comparing adolescents in Greece and Finland, there were higher level of anxiety and depression in Greece but the overall conclusion was that differences between these Northern and Southern regions were small [33]. A study comparing YSR-scores from seven countries also concluded differences were small between countries [34]. Being cross-sectional, our study cannot explain the reporting of higher mental distress by Cambodian youth compared with youth from Nicaragua. It is possible that the fairly recent period of collective trauma, experienced by the parents and grandparents of youth, may contribute to the mental health problems in Cambodia. Several studies have shown that mental health problems are common among the survivors of genocide and that mental health problems are likely to be transmitted from one generation to the next through various mechanisms [35].

Thirdly, mental distress as related to suicidal expressions showed a different pattern in Cambodia and Nicaragua. Despite higher level of mental distress in Cambodia, the prevalence of serious suicidal expressions was not different between the two countries. Furthermore, almost all mental syndromes were strongly associated with serious suicidal expressions in Nicaragua but few associations were identified in Cambodia. We believe that cultural differences between these two countries with otherwise similar socio-economic conditions might play a role to prevent high level of mental distress from increasing serious suicidal expressions. Religion has been suggested to act as a protective factor, even though some studies show a mixed picture [6,36] Despite the fact that the Catholic church in Nicaragua strongly condemns suicidal behavior whereas Buddhism seems to have a more tolerant view, serious suicidal expressions are as common in Nicaragua despite lower mental distress. One explanation could be that Nicaraguan culture is more secularized than Cambodia where traditional values still have a stronger influence on young people.

Fourthly, there are gender and country differences, where Nicaraguan boys exposed to suicide were almost nine times more likely to report own serious suicidal expressions while there was no significant association for Cambodian boys. Association between exposure to suicide and own suicidal expressions have been reported from many countries [1]. For example, in Nicaragua this has been confirmed in a community based study [10]. In most previous studies the association has been reported to be more evident among girls. For Nicaraguan boys depressive symptoms and exposure were associated with serious suicidal expressions whereas for Cambodian boys they were not.

### Limitations

There are various definitions on what is meant with culture and cross-cultural research [3]. Even though this study does not take the cultural context into consideration in the analyses, we add this perspective in the interpretation of our results and we suggest that this makes it legitimate to label the study "cross-cultural".

Small sample size might have limited the results. The research instruments, ATTS and YSR, were developed in high income countries and may not measure the same constructs in the same manner in low-income countries such as Nicaragua and Cambodia. The nuances of emotions and psychological constructs are challenges to fathom particularly when the instrument is translated into different languages for cross-cultural comparison [3]. However, both instruments have been used in diverse settings and found valid [26,27,29,30]). Another important consideration is that the study deals with young people attending school and findings cannot be generalized to those outside the educational system.

## Conclusion

This study lends weight to the argument that both universality as well as cultural specificity characterizes serious suicidal expressions as suggested by several researchers [37]. Whereas prevalence shows less variation between cultures, this study shows that associated factors behave differently, which we believe calls for different preventive approaches. Qualitative research approaches are warranted to further explore possible cultural bound mechanisms behind suicidal expressions among young people.

## Competing interests

The authors declare that they have no competing interests.

## Authors' contributions

CMO and BJ were involved in planning the study, organized data collection, performed analyses and drafted the manuscript. GK planned the study and supervised analyses and drafted the manuscript. KD planned the study and drafted the manuscript. All authors read and approved the final manuscript.

## Pre-publication history

The pre-publication history for this paper can be accessed here:

http://www.biomedcentral.com/1471-244X/12/28/prepub
